# Detection and Quantification of Alprazolam Added to Long Drinks by Near Infrared Spectroscopy and Chemometrics

**DOI:** 10.3390/molecules27196420

**Published:** 2022-09-28

**Authors:** Claudia Scappaticci, Stella Spera, Alessandra Biancolillo, Federico Marini

**Affiliations:** 1 Department of Chemistry, University of Rome “La Sapienza”, Piazzale Aldo Moro 5, 00185 Rome, Italy; 2 Department of Physical and Chemical Sciences, University of L’Aquila, Via Vetoio, 67100 L’Aquila, Italy

**Keywords:** near infrared (NIR) spectroscopy, alprazolam, chemometrics, partial least squares (PLS) regression, partial least squares discriminant analysis (PLS-DA), drug-facilitated sex assault (DFSA)

## Abstract

In the present work, a fast, relatively cheap, and green analytical strategy to identify and quantify the fraudulent (or voluntary) addition of a drug (alprazolam, the API of Xanax^®^) to an alcoholic drink of large consumption, namely gin and tonic, was developed using coupling near-infrared spectroscopy (NIR) and chemometrics. The approach used was both qualitative and quantitative as models were built that would allow for highlighting the presence of alprazolam with high accuracy, and to quantify its concentration with, in many cases, an acceptable error. Classification models built using partial least squares discriminant analysis (PLS-DA) allowed for identifying whether a drink was spiked or not with the drug, with a prediction accuracy in the validation phase often higher than 90%. On the other hand, calibration models established through the use of partial least squares (PLS) regression allowed for quantifying the drug added with errors of the order of 2–5 mg/L.

## 1. Introduction

Alprazolam is an active pharmaceutical ingredient (API) present in different psychotropic drugs belonging to the class of benzodiazepines. Benzodiazepines are a class of anxiolytics effective in both the acute and chronic treatment of patients with sustained or recurrent anxiety attacks. In addition to their anxiolytic effects, benzodiazepines produce sedative, hypnotic, anesthetic, anticonvulsant, and muscle relaxant effects. They can alter motor control and enhance the effects of other substances, such as alcohol. One of the most well-known pharmaceutical preparations containing alprazolam is Xanax^®^, which is sold under this name both in Europe and in the USA. The Xanax^®^ formulation is conceived for immediate effect and relief is almost instantaneous, but its effect is short-lived. In recent years, its use for the preparation of “spiked” cocktails for recreational purposes, or, in even worse cases, for doping unaware victims, has been reported. In particular, Xanax^®^ is combined with alcoholic beverages, which increase the persistence and the concentration of Alprazolam in the blood and brain, enhancing the effects of the drug, acting on the central nervous system [1]. 

Drug-facilitated sex assault (DFSA) is a form of sexual violence against an individual made not fully cognizant by a substance that alters her/his physical and mental state, such as alcohol or drugs [2]. Several APIs (alone or mixed with alcohol) can induce loss of inhibition or consciousness, and are associated with antegrade amnesia. In DFSA, the most commonly reported drugs are GHB (γ-hydroxybutyric acid), benzodiazepines (Valium^®^, Xanax^®^, or Rohypnol^®^), antidepressants (Venlafaxine), muscle relaxants (Ciclobenzaprine), antihistamines, over-the-counter sleeping pills (Diphenhydramine), hallucinogens, and opioids. Cocktails spiked with Xanax^®^ can be easily used as a doping agent to achieve a stun effect in unsuspecting victims, because the taste of the excipients can be confused with the flavors of the long drink. As the percentage of psychotropic drug mixed with alcohol increases, the resulting effects range between not having any sensitive effect, to losing the inhibitory brakes, to loss of consciousness and loss of control of the central nervous system to the point of being unable to breathe autonomously [3]. 

Generally, in the case of suspected DFSA, different biological matrices of the victim can be analyzed, such as urine, blood, or hair [4].

The most common analytical techniques recommended for the analysis of DFSA-related samples (coupled with methodologies tailored for detecting “rape drugs” and their metabolites) are gas chromatography coupled with mass spectrometry (GC-MS) and liquid chromatography coupled with tandem mass spectrometry (LC-MS/MS) or a diode array detector (LC-DAD) [5,6].

Liquid chromatography (LC) coupled with UV–visible operating in full scan acquisition has also been proposed [7], but then the spectra need to be further compared with references to confirm drug detection.

Unfortunately, in practice, most of the samples are delivered to the analytical laboratories long after their collection, compromising the detection of low drug concentrations. To overcome this issue, MS/MS detection, such as LCMS/MS or GC-MS/MS, has been recommended due to its higher sensitivity and selectivity [7].

In recent years, together with the analysis of alternative biological matrices, the possibility of using faster and portable techniques has been investigated. In this context, a recent study demonstrated that the electrochemical determination of flunitrazepam in different degassed alcoholic beverages using plain or silk-screened graphite electrodes modified with graphene can be efficiently carried out [8].

In addition to these methods, a Raman-based solution, finalized to the detection of flunitrazepam in drinks, has also been proposed. In fact, Ali et al. demonstrated that it is possible to collect the Raman signals of beverages spiked with flunitrazepam (concentrations from 0.01 to 0.04% *w*/*v*), compare them with the reference spectra, and identify doped drinks [9]. 

Given this background, the purpose of the present work is to develop a fast and green analytical methodology to identify and quantify the presence of an adulterating drug (alprazolam, the API of Xanax^®^) within an alcoholic beverage of large consumption, namely gin and tonic, coupling near-infrared spectroscopy (NIR) and chemometrics. In particular, partial least squares (PLS) regression [10] and partial least squares discriminant analysis (PLS-DA) [11] have been exploited for quantification and detection. These strategies were chosen because they have demonstrated their suitability in similar contexts [12,13]. The final outcome of this study is manifold. In fact, if needed (e.g., in case of DFSA), the developed strategy can be applied to analyze a drink (if available) and determine whether it was (voluntarily or not) spiked with alprazolam. A further, but not less relevant, aspect is that this work can represent a feasibility study for the development of an NIR-based portable device, which can be kept in bars and night clubs, and allow customers to check their drink in case of suspected tampering.

## 2. Results and Discussion

As anticipated, the present study is two-folded involved. The first step is qualitative, aimed at discriminating pure from spiked drinks, followed by a second step, where multivariate regression was used to quantify the added drug. 

### 2.1. Discrimination of Pure and Spiked Drinks by PLS-DA 

To discriminate pure and spiked drinks, a multivariate classification strategy based on the use of partial least squares discriminant analysis (PLS-DA) on the collected NIR spectra was adopted. To validate such a strategy and evaluate its generalizability as unbiasedly as possible, particular attention was paid to the proper definition of the training and test samples. Indeed, in order to have the validation samples be as independent as possible from the training ones, it was decided to leave out all of the samples prepared with a particular brand of gin as a test set, leaving the other spectra for model building and model selection. Moreover, to check the robustness of the results with respect to the identification of the specific brands to be used for model building and validation, the procedure was repeated three times, each time selecting all of the samples prepared with a specific brand of gin as the test set and using the others as the training set. 

This means that the first model was built using all mixtures prepared with gins G1 and G2 (i.e., G1T1, G1T2, G1T3, G2T1, G2T2, and G2T3, for a total of 342 samples) as the training set and the remaining 171 samples, corresponding to the pure and spiked drinks prepared using the third gin brand (G3T1, G3T2, and G3T3) as test set. Following the same conceptual scheme, the selection of the optimal parameters and meta-parameters (number of latent variables and best spectral preprocessing) to build the final model was conducted on the training samples based on the results of a split-half cross-validation, where the two cancelation groups coincided with the two brands of gin used to prepare the mixtures (here, G1 and G2).

Similarly, a second model was built using all of the mixtures prepared with gins G1 and G3 (i.e., G1T1, G1T2, G1T3, G3T1, G3T2, and G3T3, for 342 samples) and was validated on the 171 samples prepared with gin G2 (G2T1, G2T2, and G2T3). Analogously, a third model was built on G2 and G3-based mixtures and validated on all of the samples prepared using G1. 

Successively, to further check the robustness of the approach, a similar training/test splitting scheme was adopted, this time leaving out, in turn, as the test set, all the mixtures prepared using a specific brand of tonic water. This means that a fourth PLS model was built using all of the mixtures prepared with tonic waters T1 and T2 as the training set (i.e., G1T1, G2T1, G3T1, G1T2, G2T2, and G3T2, for a total of 342 samples), and was validated on the remaining 171 samples prepared using tonic water T3 (G1T3, G2T3 and G3T3). In this case, model selection was based on the results of a split-half cross-validation, where the two cancelation groups coincided with the two brands of tonic water used to prepare the training mixtures (here, T1 and T2).

The procedure was then repeated to build and validate a fifth (using mixtures prepared with T1 and T3 tonic waters as the training and those with T2 as the test sets) and a sixth (using mixtures prepared with T2 and T3 tonic waters as the training and those with the T3 as test sets) model. 

The results obtained are summarized in Table 1, where the classification figures of merit on the six models on their respective test set samples are reported.

When considering the ability of the models to correctly recognize the class of the training samples used for model building and model optimization, a very high accuracy was obtained (in most of the cases, higher than 97%). In particular, the sensitivity for the pure class was always higher (2 to 10%, depending on the model) than that of the spiked category, irrespectively of the training/test splitting scheme adopted. The results obtained in the validation stage, i.e., when the models were applied to their respective test set samples, which are shown in Table 1, indicate that, in general, the models can be applied to new samples with a similarly high classification accuracy (almost 90% or higher in most of the cases). 

**Figure 1:**
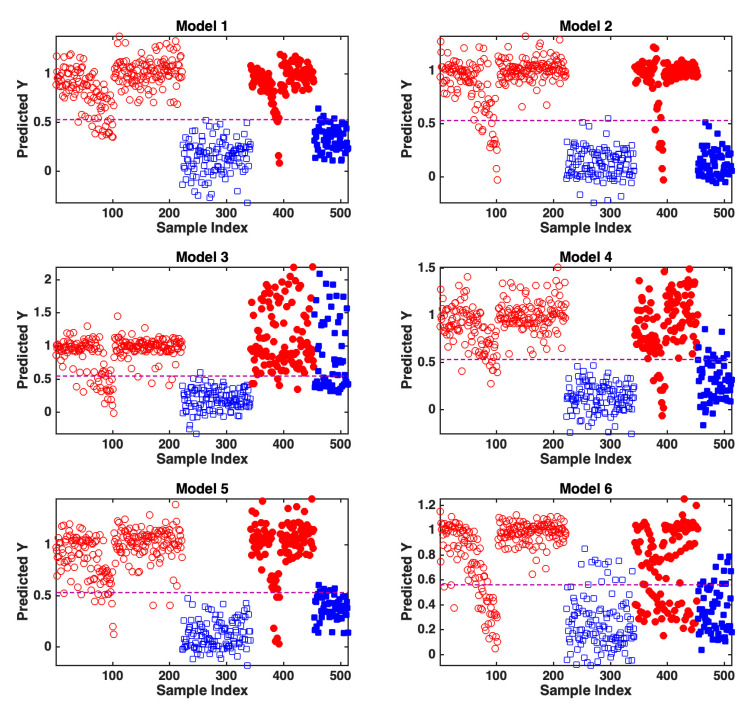
Graphical representation of the predictions of the different PLS-DA models on their respective training (empty symbols) and test (filled symbols) samples. Legend: red circles, spiked drinks; blue squares, pure drinks.

The classification ability of the different models on the training and test samples can also be graphically appreciated in Figure 1, where the predicted values of the response variable coding for class belonging are graphically displayed. In the same figure, the dashed horizontal line in each subplot represents the classification threshold: samples falling above the threshold are predicted as having been spiked, whereas those falling below are recognized as pure. 

By looking at the results in Table 1 and Figure 1, and comparing the outcomes for the training and test samples, it is possible to observe how, as already pointed out, in most of the cases, the accuracy of the predictions in the validation phase was comparable to that in the model building step, and, in general, very high. In only two cases, significantly lower correct classification rates were obtained on the test set, i.e., when either all the mixtures with gin brand 1 or those with tonic water brand 1 were used for validation; however, accuracy values higher than 70% were still obtained. 

**Figure 2:**
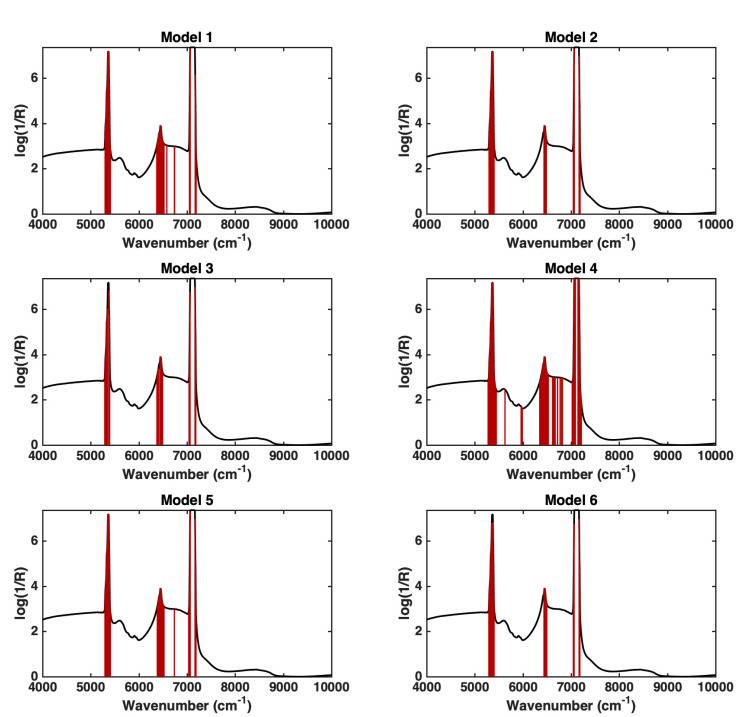
Variables identified as significantly contributing to the six calculated PLS-DA models according to the VIP analysis. For each model, the variables with VIP > 1 are highlighted as dark red bars over the mean spectrum of the training samples (black line).

The models were further inspected to evaluate which spectral variables contributed the most to the discrimination, and this was accomplished through the calculation and evaluation of the values of the variable importance in the projection (VIP) [14] indexes. Indeed, VIP is one of the indexes that is customarily considered to evaluate the variable contribution in PLS-based algorithms, especially as, because of the way they are calculated and normalized, a greater-than-one criterion can be adopted to identify those predictors that are relevantly involved in the definition of the model. Figure 2 shows the spectral regions identified as significantly contributing to each of the six calculated models according to the results of VIP analysis (i.e., the bands corresponding to VIP values greater than one). 

It is possible to observe in the figure how, irrespective of the training/test splitting adopted to build the models, most of the spectral variables identified as relevant are the same. This represents a further confirmation of the robustness of the proposed approach. In particular, the spectral intervals 5300–5400 cm^−1^, 6380–6520 cm^−1^, 7043–7062 cm^−1^, and 7166–7174 cm^−1^ are highlighted as being relevant for all of the models. The first one (5300–5400 cm^−1^, combination of O-H stretching and deformation) and the last ones (7043–7062 cm^−1^ and 7166–7174 cm^−1^, first overtone of O-H stretching) are related to the drink matrix, whereas the peak at 6380–6520 cm^−1^ is characteristic of alprazolam, as also shown in [15]. 

### 2.2. Quantification of Alprazolam by PLS 

In a second stage of the study, based on the promising results of the qualitative analysis, the possibility of quantifying the concentration of alprazolam in the spiked drinks based on NIR spectroscopy and chemometrics was also explored. In particular, PLS regression was used to build calibration models relating the spectral profile recorded on the samples to the amount of alprazolam in the drinks. 

For model building and validation, the same sample splitting schemes described in Section 2.1 for the discrimination step were adopted, i.e., leaving out, in turn, all the mixtures prepared with one gin brand or one tonic brand as the test set. Moreover, for each model, the choice of the best preprocessing and of the optimal number of latent variables was based on the results of a split half cross-validation on the training data, also carried out analogously to what is already described in Section 2.1.

The results of the PLS calibration on the six different training/test splitting schemes investigated are summarized in Table 2, where, for the sake of a better clarity, the set of mixtures selected as test set samples for each model have also been explicitly reported. 

The results reported in the table confirm what has already been seen when discussing the outcomes of the classification study: while the values of the figures of merit on the training set samples always indicate a high accuracy, the model performances on the test set individuals are comparably valid only in some of the investigated splitting schemes (here, in the case of models 1, 4, and 5). These outcomes suggest, on the one hand, that it can be possible to quantify the concentration of alprazolam in spiked drinks based on the recorded NIR spectra, but, on the other hand, they also indicate that some gin or tonic water brands have more distinctive spectral features that, if not accounted for in the model building phase, can interfere with the predictions, leading to a lower accuracy. 

**Figure 3:**
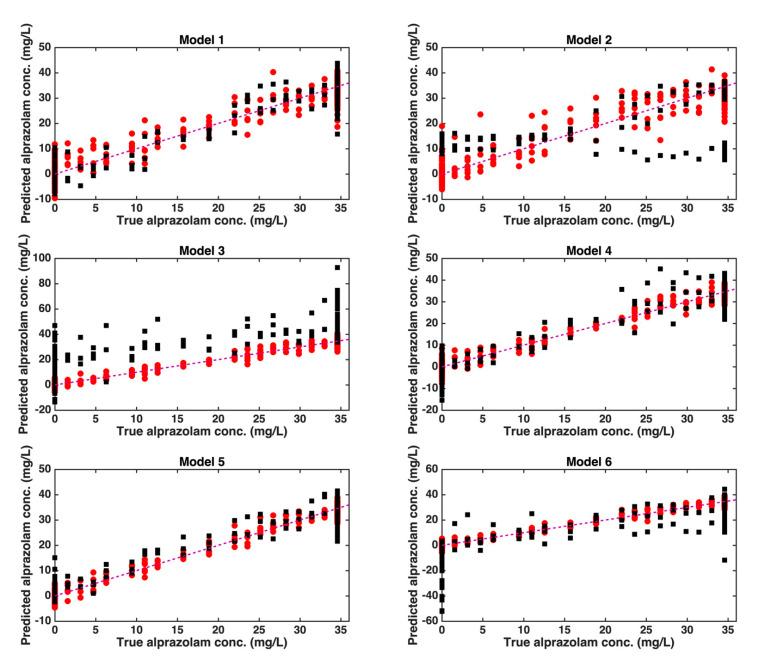
Results of PLS calibration for the quantification of alprazolam in spiked drinks. Plots of predicted vs. true values for the six calculated models. Legend: red circles, training samples; black squares, test samples.

The results obtained on the training and test samples can also be visually appreciated in Figure 3, where, for each model, the predicted concentrations of alprazolam in the mixtures are compared with their true values.

The plots in the figure highlight how, in the case of splitting schemes 1, 4, and 5, the prediction accuracy on the test samples is comparable to that observed for the training set. On the other hand, in the case of model 6, the not completely satisfactory results are mostly due to the inaccuracy in predicting the absence of the drug in the drink (the highest absolute errors are for the samples where the alprazolam concentration is zero. Lastly, model 2 and, in particular, model 3, suffer from the obvious presence of a relevant systematic error when applied to the test samples. 

For all of the models, a multivariate limit of detection was estimated according to the approach proposed by Allegrini and Olivieri [16], and the values obtained were found to be in the range of 0.4 to 0.9 mg/L. Considering that the volume of a long drink serving is usually about 200 mL, such LOD values would allow for detecting the addition of 0.08 to 0.18 mg of alprazolam, which is well below the minimum dose considered as metabolically relevant (0.5–1.0 mg) [17], indicating that the proposed approach is suitable for the detection of the fraudulent addition of API to long drinks.

Analogously to what has already been done when interpreting the classification models, also in the calibration context, VIP scores can be calculated and inspected in order to identify the variables contributing the most to the definition of the regression relation. 

**Figure 4:**
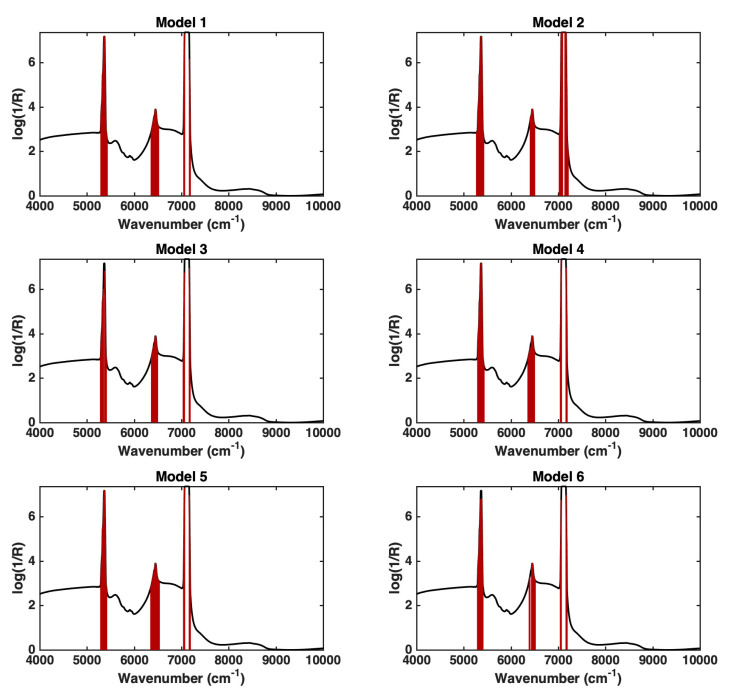
Variables identified as significantly contributing to the six calculated PLS models according to the VIP analysis. For each model, the variables with VIP > 1 are highlighted as dark red bars over the mean spectrum of the training samples (black line).

Accordingly, Figure 4 shows, for each calculated model, the spectral regions identified as significantly contributing to the regression on the basis of their VIP index. 

By looking at Figure 4 and comparing it with Figure 2, it is evident how the spectral regions identified as significant for the quantification of the drug in the drinks are practically the same, resulting in being relevant for the discrimination between pure and spiked drinks. This is further confirmation of the soundness of the proposed approach and provides an additional validation of the results obtained. 

## 3. Materials and Methods

### 3.1. Sample Preparation 

For the preparation of all of the gin and tonic samples, both spiked and pure, it was decided to use three different commercial brands of gin (G1, G2, and G3) and three diverse brands of tonic water (T1, T2, and T3), and preparing all of the possible combinations of gins and tonic water, corresponding to mixing a brand of gin with a type of tonic water. This resulted in nine series of mixtures, here labeled as G1T1, G1T2, G1T3, G2T1, G2T2, G2T3, G3T1, G3T2, and G3T3. All of the beverages were bought in Italian supermarkets.

All of the gin tonic samples were prepared by mixing the selected brands of gin and tonic water at a ratio of 1:2 (*v*/*v*). For each of the nine combinations of gin and tonic water brands, 20 samples of pure (unspiked) gin tonic were prepared, resulting in a total of 180 genuine specimens. On the other hand, for each combination of the gin and tonic water brands, 37 spiked samples with an alprazolam concentration ranging from 1.6 to 34.6 mg/L were prepared by adding to the drink the proper amount of Xanax^®^ (oral drops; alprazolam concentration 0.75 mg/mL, Ph. Eur.). Accordingly, a total of 333 spiked samples were prepared.

### 3.2. NIR Spectra Collection

Near infrared spectra were acquired using a FT-NIR Antaris II instrument (Thermo Scientific Inc., Madison, WI, USA), equipped with a tungsten–halogen source and an InGaAs detector. The spectra were acquired in transmission mode in the range 10,000–4000 cm^−1^, accumulating 16 scans at a nominal resolution of 4 cm^−1^. Operationally, 0.9 mL of sample was pipetted into a polypropylene vial (8 mm diameter), which was then placed into the instrumental compartment for spectral acquisition. The NIR signals were acquired and exported for further chemometric processing by means of the instrumental software Result (Thermo Scientific Inc., Madison, WI, USA).

### 3.3. Chemometric Methods

#### 3.3.1. Partial Least Squares

Partial least squares [10,18,19] is a regression approach, which allows for approximating the relationship between one or more dependent variables (***Y***) and a set of predictors (***X***). PLS is a very efficient regression tool, and it can be profitably used for the quantification of analytes in mixtures based on spectroscopic measurements due to its ability to cope with many correlated variables and with problems where the number of training sample is lower than that of the predictors.

For a single response case (**y**) [20], the algorithm iteratively extracts orthogonal ***X***-scores (***T***), presenting the highest covariance with **y**:

(1) T=XR

where R is a matrix of weights allowing for the direct calculation of scores from the predictor matrix ***X***. Once the desired number of components (F) are extracted, the response is regressed onto the scores according to the following:

(2) $$\hat{y}=Tq$$

where the predicted responses are collected into the vector ŷ while ***q*** is the regression coefficients expressed in terms of the scores (Y-loadings). Substituting Equation (1) into (2), it is possible to obtain a regression model where the predicted responses are directly expressed in terms of the measured variables:

(3) $$\hat{y}=Tq=XRq=Xb$$

where the regression coefficient vector is given by

(4) b=Rq

As the predictions depend on the selected number of components (also called latent variables (LVs)), this parameter has to be optimized during the model selection stage. This is usually done by selecting the number of latent variables that lead to the lowest prediction error in cross-validation, and such an approach was also followed in the present study.

#### 3.3.2. Partial Least Squares Discriminant Analysis

Partial least squares discriminant analysis (PLS-DA) [11,21,22] is a discriminant technique that exploits the advantages of the PLS algorithm to deal with classification problems involving predictor matrices with many variables, which can also be highly correlated.

The possibility of using a regression algorithm such as PLS to deal with classification problems relies on finding a suitable way for coding the response variable **y**, so as to account for the class membership. In particular, in the case of a two-class problem, such as the one considered in the present studies (where the categories involved are “pure” and “spiked” drinks), the class belonging is encoded in a dummy binary **y** vector, in which 1 codes for the one of the classes (here, “spiked”) and 0 for the other (here “pure”). Then, a PLS regression model relating the ***X*** matrix containing the instrumental responses and the binary-valued **y** matrix encoding class membership is built, and classification is then carried out on the basis of the predicted values of the response. However, differently than their target values, which are binary-coded, predictions are real-valued and therefore a classification rule is needed in order to be able to assign the samples to one or another category. In a two-class problem, this corresponds to identifying a threshold value for the response, so that if the predicted response is higher than that threshold, the sample is assigned to the class labeled as 1, while if it is lower, the sample is predicted as belonging to the class coded as 0. In the present study, the classification threshold was defined by applying linear discriminant analysis (LDA) [23] to the vector of the predicted responses. 

As PLS-DA is based on the PLS algorithm, in this case, it is also necessary to define the optimal number of components to be included in the model. In the present study, this was accomplished by selecting the model complexity leading to the lowest classification error in cross-validation. 

## 4. Conclusions

The presence of drugs in beverages, both alcoholic and non-alcoholic, is a problem of significant importance and, with the aid of quick detection approaches, numerous rape cases could be solved. The aim of this study was to verify the possibility of identification and quantification of the fraudulent addition of these drugs, more specifically alprazolam, to an alcoholic drink, gin and tonic, by means of the coupling of near-infrared spectroscopy and chemometric techniques.

The approach used was both qualitative and quantitative, as models were built that would allow for highlighting the presence of aprazolam with a high accuracy, and to quantify its concentration with, in many cases, an acceptable error.

Thanks to the use of PLS-DA, it was possible to build models that allowed the samples to be classified as spiked or not, obtaining correct prediction rates, in the validation phase, often higher than 90%. In parallel, with the further use of the PLS algorithm, it was possible to build calibration models that allowed for quantifying the drug added with errors of the order of 2–5 mg/L.

In conclusion, the proposed method allows for the rapid and non-destructive detection of the addition of alprazolam to gin and tonic samples, and the quantification of the added drug with a good degree of accuracy. At the same time, the proposed validation scheme has made it possible to highlight that there may be a non-negligible effect of the matrix on the possibility of making correct predictions; therefore, in order to obtain more accurate predictions, it would be appropriate to extend the set of samples to others gin and tonic water brands. 

Furthermore, the results obtained suggest that this approach could also be extended to the identification of the addition of other rape drugs to alcoholic and non-alcoholic beverages.

## Figures and Tables

**Table 1:** Classification results of the six calculated PLS-DA models on their respective test set samples.

Model	Mixtures Used as Test Set	Pre-Treatment *	LVs *	Accuracy (%)	Average % CCR *	Sensitivity (%)	
						Spiked	Pure
1	G3T1 G3T2 G3T3	MC	13	95.32	94.86	96.40	93.33
2	G2T1 G2T2 G2T3	MC	10	95.32	96.40	92.79	100.00
3	G1T1 G1T2 G1T3	MC	6	78.36	71.08	95.50	46.67
4	G1T3 G2T3 G3T3	D1+MC	21	88.89	88.00	90.99	85.00
5	G1T2 G2T2 G3T2	SNV+MC	16	92.98	92.30	94.59	90.00
6	G1T1 G2T1 G3T1	MC	3	71.35	73.72	65.77	81.67

* MC, mean centering; SNV, standard normal variate; D1, first derivative; % CCR, correct classification rate (%); LVs, number of latent variables.

**Table 2:** Results of PLS calibration for the quantification of the alprazolam concentration in spiked drinks.

Model	Mixtures Used as Test Set	Pre-Treatment *	LVs *	Calibration (Training Set)			Validation (Test Set)		
				RMSEC *	Bias	R^2^	RMSEP *	Bias	R^2^
1	G3T1 G3T2 G3T3	MC	14	4.0	0.0	0.9321	4.9	0.5	0.9017
2	G2T1 G2T2 G2T3	D2+MC	14	4.2	0.0	0.9270	12.7	−0.9	0.3352
3	G1T1 G1T2 G1T3	MC	6	2.6	0.0	0.9730	19.6	−15.1	−0.5879
4	G1T3 G2T3 G3T3	SNV+MC	24	2.5	0.0	0.9748	5.8	−0.1	0.8596
5	G1T2 G2T2 G3T2	SNV+MC	25	2.1	0.0	0.9810	5.1	−0.7	0.8919
6	G1T1 G2T1 G3T1	MC	22	2.1	0.0	0.9820	13.0	6.3	0.2965

* MC, mean centering; SNV, standard normal variate; D2, second derivative; RMSE, root mean square error of calibration; RMSEC, root mean square error of prediction; LVs, number of latent variables.

## Data Availability

Not applicable.

## Sample Availability

Samples of the compounds are not available from the authors.
